# Patients' requests for radiological imaging: A qualitative study on general practitioners' perspectives

**DOI:** 10.1111/hex.13849

**Published:** 2023-08-16

**Authors:** Lizzie De Silva, Melissa Baysari, Melanie Keep, Peter Kench, Jillian Clarke

**Affiliations:** ^1^ Discipline of Medical Imaging Science, Sydney School of Health Sciences, Faculty of Medicine and Health The University of Sydney Sydney New South Wales Australia; ^2^ Biomedical Informatics and Digital Health, Charles Perkins Centre D17 Faculty of Medicine and Health Sydney New South Wales Australia; ^3^ Sydney School of Health Sciences Faculty of Medicine and Health Camperdown New South Wales Australia

**Keywords:** consumerism, doctor–patient relationship, general practitioner, imaging, internet, patient‐requested radiology

## Abstract

**Background:**

With the increasing availability of information, patients are becoming more informed about radiology procedures and requesting imaging studies. This qualitative study aims to explore factors that influence general practitioners' (GPs) decisions to fulfil patient requests for imaging studies during clinical consultation.

**Methods:**

Semi‐structured interviews were conducted with 10 GPs working across five private medical centres in Northwest Sydney. Conventional content analysis was used with emergent themes to identify GPs perspectives.

**Results:**

Six themes stood out from the interviews with GPs fulfilling patient requests for imaging studies. They included four pertaining to patient factors: patient expectations, ‘therapeutic scans’, ‘impressive labels’ and entitled. Two further themes pertained to the GP perspective and included defensive medicine, and ‘new patients’. Requests are fulfilled from anxious or health‐obsessed patients, with GPs worrying about litigation if they refuse. However, GPs decline requests from patients with entitlement attitudes or during first visits.

**Discussion:**

The findings suggest that GPs struggle to balance their responsibilities as gatekeepers of imaging with patients' expectations of request fulfilment. Clear guidelines on the appropriate use of diagnostic imaging and its limitations could help patients understand its proper use and ease anxiety. Additionally, education and training for GPs could help them manage patient expectations and provide appropriate care.

**Patient Contributions:**

Patients, service users, caregivers, people with lived experiences or members of the public were not directly involved in the design, conduct, analysis or interpretation of the study. However, our study was conducted in primary care facilities where the GPs were interviewed about patients' requests for diagnostic imaging based on their own initiatives. GPs' perspectives in managing patient expectations and healthcare utilisation were explored within the Australian Medicare system, where medical imaging and image‐guided procedures come at little to no cost to the individual. The study findings contribute to a better understanding of the challenges faced by GPs in dealing with patient consumerism and requests for diagnostic imaging, as well as factors influencing request fulfilment or denial. Insights gained from this study may inform future research about delivering patient‐centred care within a similar context.

## 1 INTRODUCTION

In Australia, patients are required to have a general practitioner (GP) referral to access imaging services. GPs face several conflicts in assuming this gatekeeping role. They must negotiate patient requests for imaging while maintaining a good rapport with patients. Further, they need to protect patients from the harms of medical exposure, while limiting overuse of healthcare resources.^1^ This authoritative or paternalistic role requires expert judgement when deciding what evidence‐based tests and procedures are appropriate for achieving patients' clinical diagnoses.^2^, ^3^

However, with patients' increasing use of the internet,^4^ digital platforms,^5^ and accessibility to personal online health records^6^ has shifted the traditional role of GPs in the healthcare system.^7^, ^8^ With the increased availability of health information online, patients can now more easily access and learn from information about medical conditions and health‐related issues independently,^4^ and can consider themselves well‐informed health consumers.^3^ This has contributed to the rise of patient consumerism. As such, GPs increasingly encounter patients who request studies, and rather than relying on their expert guidance, challenge the GPs role as gatekeeper.^9^

One aspect of patient consumerism is the demand for patient‐centred care.^10^ Patients expect GPs to acknowledge their needs, perspectives and understand the complex psychosocial aspects of the illness process.^11^ However, there are concerns associated with patient‐centred care.^12^ Some argue that the increased emphasis on meeting patient demands may drive healthcare costs higher.^10^ Moreover, GPs may feel pressured to order unnecessary tests or procedures to alleviate their own anxiety and fear of ligation.^13^, ^14^ This could result in the overutilisation of healthcare resources and potential financial burden.

Thus, with the increasing use of imaging services,^15^ concerns have been raised over the appropriateness of the tests and procedures requested by patients.^16^ While patients have become more knowledgeable and self‐directed, particularly those in higher socioeconomic settings,^17^, ^18^ often medical consultations have become a means to secure a self‐initiated request for a medical imaging study. This challenges the traditional role of GPs as the primary source of medical advice and decision‐making.

Overall, the evolving landscape of patient consumerism, driven by increased internet access and patient‐centred expectations, necessitates a response from GPs. The perceived pressure on GPs to order diagnostic tests has been explored by Griffith et al.^19^ with additional studies centred on patient–GP communication, concerns over unnecessary expenditure in imaging^20^ and avoiding imaging overuse.^21^ However, there is limited research on how GPs personally respond to patients requesting radiological studies. Our study aimed to explore with GPs, the patient–GP relationship factors that influence granting patient requests and the relationship between request fulfilment and patient characteristics. Specifically, the research questions were: What factors facilitated GPs to meet or decline requests, and what patient characteristics enabled (or not) request fulfilment. The University of Sydney ethical review board approved this study.

## 2 METHODS

### 2.1 Recruitment

GPs in private medical centres with attached imaging facilities throughout metropolitan North‐Western Sydney, Australia were initially contacted via an advertisement on the medical centres' intranet. Those interested indicated their willingness to participate, and the researcher then followed up with each doctor for an interview. A qualitative approach such as interviews lends detailed and nuanced understanding of the research questions with emergent themes adding meaning to the data and contributing to theory development.^14^ With such a methodology, participants' experiences and perspectives within primary care facilities were explored through the richness and complexity of the data. Written consent was obtained before commencing. Semi‐structured interviews were conducted face‐to‐face (*n* = 6) or on Zoom (*n* = 4). Interviews were between 20 and 40 min (*M* = 25, SD = 9.33) between August 2022 to September 2022.

Participants provided demographic information such as their length of time in practice, and their experiences of patients requesting radiological referrals. The interview was then guided by open‐ended questions developed through an extensive literature search^10^, ^13^, ^21^, ^22^, ^23^, ^24^ and expert consultation with the imaging department's healthcare professionals, for example, radiologists, specialists and physiotherapists. These included the types of exams frequently requested, the characteristics of patients, their concerns and demographics of patients likely to request referrals. Participants were also asked about their professional relationship with the radiologists they refer to, and any access to and/or the availability of specific guidelines for GPs regarding tests/procedures used in radiological studies. Additionally, interviewees were asked about any steps taken to avert unnecessary requests, particularly when dealing with aggressive or overbearing patient behaviour. All interviews were conducted by the first author (L. D. S.) who audio‐recorded it on her iPhone. As soon as practicable and by the end of the day, she transcribed the interview verbatim allowing full and immediate immersion into the data. All identifying components were removed as the text was typed and then the audio recording was permanently deleted from the iPhone immediately, in compliance with ethical approval.

### 2.2 Data analysis

Transcriptions were initially read and reread by the first author (L. D. S.) searching for meanings and patterns used in thematic analysis, allowing immersion into interviewees' perspectives through familiarisation of the depth and breadth of data.^25^ The transcribed data were organised as codes under generated themes using NVIVO release 1.7(1533) software package. Using such an analytical tool allowed for flexibility as it adapts to broader textual sources such as interviews by organising, categorising and quantifying textual information through explicit coding rules and procedures for future replicability.^15^, ^26^ In our study, codes representing key thoughts or themes were derived in alignment with research objectives.^26^ Each code was subsequently separated by categories and subcategories with names that described the nature of each derived code for thematic analysis.^26^ The first author (L. D. S.) coded all the interviews, with an experienced qualitative researcher supervising the coding process. A second team member (J. C.) looked through the interview data to generate additional themes or review the ones generated by the first author (L. D. S.). Themes and subthemes were sent to the research team for consensus. All came to an agreement on the themes derived.

## 3 RESULTS

In total, 10 GPs aged 35–73 years (*M* = 56.6 years, SD = 14.1 years) were interviewed until no new themes were generated, reaching saturation. They had 5–40 years (*M* = 23.3, SD = 2.6 years) experience in practice, and identified as either male (four) or female (six).

The analysis revealed six themes around factors surrounding GPs meeting patients' requests for radiological referrals shown in Table 1 below, with explanations for each generated theme.

**Table 1:** Themes generated from the interviews with frequency in percentage.

Theme	Explanation and comments	Percentage of participants commenting on theme (*n* = 10)
Expectations	There is an assumption by patients that in requesting diagnostic imaging they are being proactive in addressing their health concerns. Moreover, this assumption is associated with prior radiological experience.	80%
‘Therapeutic scans’	A phenomenon experienced by GPs is where they may feel compelled to oblige patients' requests for certain tests or treatments as a form of reassurance, even if they do not necessarily agree with the nature of those requests. This behaviour is often driven by the desire to satisfy patients and provide them with a sense of reassurance, comfort and closure with their health concerns.	70%
‘Impressive Labels’	These concerns were raised by GPs regarding patients' accessibility to diagnostic information from prior radiological scans or reports. When patients have access to their past medical records, including radiological imaging results, there is a potential for them to misinterpret their current symptoms by comparing them to their previous experiences or those of others they know such as family members or friends.	60%
Defensive medicine	In our study, if a patient presents with symptoms indicative of a fracture or any serious medical condition, the GP faces a difficult decision. If the GP decides not to order the requested test and the condition is later diagnosed, there is a possibility that the patient or their family may pursue legal actions against GPs.	30%
New patient	GPs had very strong views about individuals demanding studies on their first visits. Without knowing patients' medical history and previous diagnostic tests, it becomes challenging for GPs to determine the appropriateness of specific medical studies. GPs, however, were more obliging to those in long‐term Patient–GP relationships.	60%
Entitled	Most GPs were concerned Medicare subsidised studies allowed greater accessibility to imaging. Such access may lead some patients to perceive it as their ‘right’ to receive such diagnostic tests whenever they ask regardless of its necessity.	70%

Abbreviation: GP, general practitioner.

### 3.1 Expectations

GPs explained that patients were generally aware of imaging services being readily available and that such services were funded under Medicare at no cost to them.

Half of my patients ask [for scans] because they know that radiology is free in this country. (Male, 55)

Such freely available imaging services meant there was an expectation for doctors to fulfil patients' requests. Participants highlighted that such expectations generally came from those who were more health literate, had employment and with higher socioeconomic status. These individuals, GPs explained, were well informed about their symptoms and illnesses and indicated the need to be actively involved in their health by requesting scans and interventional procedures using imaging. Many had concerns over returning to work as motivators.

Some struggling with persistent pain would ask for X‐rays because they were familiar with these types of scans and know that it is available to them. You know they've put up with back pain so much they want to fix it quickly so they can get back to work. (Male, 73)

Definitely those in the Type A personality. Those who are very motivated and want to be informed particularly in skeletal injuries wanting to know how long they will be out [of work] for? Whether it is 4–6 weeks. Especially if they have an event and want to take care of their injuries as soon as possible. (Female, 52)

### 3.2 ‘Therapeutic scans’

Another recurrent theme was that interviewees felt some imaging tests requested by their patients have a ‘therapeutic’ value through easing their anxiety, for example, related to possible pathologies.

I find X‐rays have become a therapeutic requirement, [and] that their [patients'] problem often dissipates once the request is given. (Female, 40)

GPs pointed out that these individuals had experienced illnesses where radiological services resulted in perceived benefits. Having had a prior positive experience with medical imaging, and experiencing anxiety over their illness, prompted patients to request ‘therapeutic scans’. Some GPs considered that individuals wanting therapeutic scans were often fixated with their health and self‐diagnosed without formal clinical assessments from health professionals.

These individuals are increasingly obsessed with their health, they are particularly anxious and want reassurance with X‐ray. (Female, 70)

Every day we have people coming in and asking if they could have an X‐ray you know. We can clearly see they don't need it and yeah and saying they possibly have a fracture. I try and do a clinical examination and avoid X‐ray and CT especially with kids. But you know they push us just to get that X‐ray or CT because you know the last time, they had it [X‐ray], they found a fracture or something like that you know, and they insist on having one now because they think it is the same problem. (Male, 60)

GPs also indicated that health anxiety prompted patients to search for their symptoms and illnesses online. Some participants mentioned that government‐sponsored advertising increased patient awareness by encouraging patients to check for bowel, breast or prostate cancer. GPs quickly pointed out that although such media publicity and social media platforms provide useful dissemination of health information, they also initiated a level of anxiety.

### 3.3 ‘Impressive labels’

Therefore, in potentially losing their gatekeeper role, participants agreed that patients needed appropriate guidance discerning the most appropriate test or procedure. Although GPs observed that patients were indeed well informed, they lacked critical understanding on the limitations and merits of various tests and procedures. For example, using X‐rays for acute bronchitis is not beneficial but it is useful for pneumonia where the effects are visible on X‐rays.^27^

Often, they have the wrong impression about what a test will exclude or not exclude. (Female, 60)

Prior patient exposure to radiological reports using phrases such as ‘degenerative disc disease’, a common occurrence found in the aging population,^28^ were reported as a prompt to seek further scans. One participant observed that these phrases were ‘impressive labels’ considered significant by patients.

I mean to be honest; a lot of imaging services will give you a label just to find things. Impressive labels on things that are not significant. It's what patients are looking for and it is what they want to hear. (Male, 73)

So, most anxious patients, what they do is they just Google their symptoms. Yes. And then they see differential diagnosis and bowel cancer is high up on the list, they are [then] convinced that they have bowel cancer. (Femal*e*, 40)

### 3.4 Defensive medicine

GPs expressed concerns about the potential threat of litigation if they denied patients' imaging requests even when they deemed the request inappropriate. Three out of the 10 participants indicated the need to practice defensive medicine.

Because … if they [patients] do not do physiotherapy, [and] they return to sporting activity and if it shows later on X‐rays that there was a chip or fracture, they will point the finger at the doctor—that he didn't do the X‐ray. That's the reason I would be more cautious. (Male, 55)

However, most GPs agreed that within a long‐term, established GP–patient relationship, GPs were likely informed about patients' personalities, expectations and medical history and could engage in appropriate clinical negotiations.

### 3.5 New patient

While participants were concerned about avoiding potential litigation, most agreed that their regular patients, those in long‐term GP–patient relationships, would likely comply with their guidance. In other words, if they felt certain tests or procedures were inappropriate and they explained why, they believed their regular patients would likely comply with recommendations. This was not viewed to be the case for the unfamiliar patient who was seeing that GP for the first time.

Yeah, difficulty is with patients who I don't know. They are walk‐in patients; They say, I want to have an X‐ray of my whatever. The first thing I do is not [get into a] confrontation. First thing I say, oh, excellent. Of course, we can organise that. Can I know the reason? Can you please give me some details? Then, you start talking and you start looking and at some stage you decide whether they actually need the X‐ray or not. (Male, 55)

In particular, participants noted that these patients ‘tend to be noncompliant and less receptive to guidance often seeking the last available appointment’ (Female, 40), giving the GP limited opportunity to explore the reasons for the request and building sufficient rapport to advise if the request is not clinically recommended.

Participants perceived that those patients new to the GP used medical consultations as a formality to secure a referral request, and GPs were generally reluctant to comply with such requests.

They [new patients] probably don't even trust the doctor that much… they will trust the CT or ultrasound scans better than trusting the GP. (Female, 40)

Patients say, oh you're not my regular GP, but can you do this for me? It is that sort of attitude that we sometimes get from some patients …they insist [saying] my GP will give me what I want, or they usually give me this and that… so I say, Then go [and] see your GP.

GPs generally require more time with new patients to familiarise themselves with patients' medical history. The process of familiarisation often lengthens consultation and disrupts workflow.

But sometimes it was easier and quicker just to accede to them, if they want back X‐rays. Well, it's not a good idea. But you know, sometimes you did it, because you had a line‐up of people waiting, and that was going to take a long time to explain, and you weren't going to convince them [otherwise] anyway. (Male, 73)

Moreover, GPs explained that new patients making such requests ‘have made up their mind and if you [GP] still are hesitant, they most likely find another doctor who will give them their request’ (Female, 42).

### 3.6 Entitled

With most radiological studies covered under the taxpayer‐funded Medicare Benefit Scheme (MBS), patients felt entitled to scans and procedures. MBS describes the types of services that can be subsidised under Medicare (http://www.mbsonline.gov.au). GPs found adhering to the MBS guidelines challenging, for example, one GP stated that many of her patients wanted computer tomography (CT) calcium scoring after the death by heart attack of a famous Australian cricketer. The associated publicity explained that CT‐measured calcium deposits in coronary arteries indicate an increased risk of heart disease.

CT calcium score requesting was increased so that was significant. In the last six months a lot of patients were prepared to go to cardiologists, but they want to be proactive themselves. They [wanted] to be reassured by scores of 0. I try and stick to the guidelines as much as possible. (Female, 52)

Moreover, if patients had a strong family history of certain illnesses, they were more than likely anxious and want medical imaging to reassure themselves they were disease free.

My patient whose sister had ovarian cancer wanted a pelvic ultrasound. So, it's more for reassurance. Or they will come and ask for bloods and want everything done and you start to pick up unnecessary things that you now need to treat. (Female, 42)

Whether due to media publicity or anxiety over family history, adherence to Medicare and radiological guidelines was challenging for the GPs. As a result, they often found themselves in conflicting situations of easing patients' anxieties while attempting to enforce existing guidelines.

**Figure 1:**
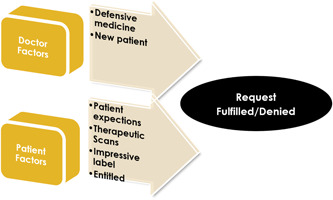
Patient–doctor factors in fulfilment or denial of requests.

In summation, four themes centred around patient factors: patient expectations, ‘therapeutic scans’, ‘impressive labels’ and entitlement. Two themes centred around the GP's perspectives, including defensive medicine, and ‘new patients’. This is portrayed in Figure 1.

## 4 DISCUSSION

This study aimed to understand factors influencing GPs' responses to patients' requests for imaging. We found that GPs were more likely to grant requests from health‐literate, anxious patients with prior radiological experience who came with explicit expectations. Patients with prior experience with radiological services perceived benefits by requesting referrals for studies, however, with a limited understanding of complex radiological terms, patients may misunderstand pertinent information. Moreover, GPs believed that anxiousness was further emphasised through public health information dissemination and that misunderstanding may lead patients to request radiological studies. However, this was problematic, as they lacked critical understanding of the merits and limitations of tests and procedures. Further, new patients were more likely to request radiological studies, and GPs often complied due to time constraints and a perceived threat of possible litigation.

In Australia, Medicare covers many medical imaging and imaging‐guided procedures with little to no cost to the individual,^26^ leading patients to expect GPs to comply with requests. The GPs in our study acknowledged the challenge of denying requests in established GP–patient relationships and emphasised the importance of focusing on overall patient outcomes. This is consistent with Carlsen et al.,^29^ who found that denying a patient's request in a well‐established GP–patient relationship is difficult, and GPs felt uncomfortable doing so. However, imperatives such as radiation safety and the risk of overservicing due to incidental findings from unnecessary imaging prompted concerns in our GPs. Mendelsohn^30^ emphasised the need to justify unnecessary imaging requests for overall patient benefit. Fenton et al.^31^ stressed the value of several interventions to reduce the occurrence of low‐value diagnostic requests, including better patient–GP communications and implementing referral guidelines for healthcare practitioners during such situations. Walderhaug et al.^21^ found, as in our study, that patients' trust in GPs negates unnecessary requests, and such trust is usually enabled through long established GP–patient relationships.^21^

However, as patients become more health literate and have greater access to medical records,^11^ they are increasingly aware of medical terminology and labels. Patients may be concerned by seemingly impressive medical terms, such as ‘degenerative disc changes’, despite these being considered normal for older adults.^28^ Brodersen et al.^32^ ^(p.1‐3)^ defines this as, ‘making people “patients” unnecessarily, by identifying problems that were never going to cause harm or by medicalising ordinary life experiences through expanded definitions of diseases’. These authors agree this can result in unnecessary medicalisation, with patients seeking diagnostic imaging to confirm potential ailments. Echoing one participant in our study, patients are seeking solutions through requests on their own initiative but with little awareness surrounding reasons for their requests. While watchful waiting strategies can reduce low‐value spinal imaging,^33^ GPs often fulfil patient requests to reduce anxiety, avoid confrontation and increase satisfaction.

Moreover, GPs in this study fulfilled referrals for excessively health‐concerned patients to reassure them, calling these ‘therapeutic scans’. Part of the reason is the plethora of publicly available medical information which often exacerbates patients' anxiety and leads to unnecessary requests. Similarly, Hogue et al.^34^ points out that exposure to information about medical conditions and associated treatments in the public domain often prompts consumers to seek further information, predominantly online. Their study argued that patients exposed to information will likely expand their knowledge through online searching to better inform themselves, but that online diagnosis without medical guidance is problematic^4^ as the quality and reliability of the information is not guaranteed. As in our study, participants were concerned with public health dissemination, as some of their patients, in seeking reassurance, had requested screening for bowel or ovarian cancer. Thus, GPs in appeasing patients sometimes overlooked Medicare guidelines to ease patient concerns.

Patients' lack understanding of the true cost of studies, and that unnecessary imaging services can lead to incidental findings requiring further healthcare interventions. Those with a family history of illnesses, in response to media publicity, may request certain tests proactively, as seen in our study with patients requesting CT calcium scores. Berg et al.^35^ suggested that enforcing guidelines could reduce inappropriate requests and unnecessary imaging. However, our study, as with Gransjøen et al.,^7^ found that adherence to guidelines has caveats, including difficulty explaining guidelines in limited consultation time, lack of knowledge of established guidelines, practicing defensive medicine, and satisfying consumer‐driven patients.

### 4.1 Limitations and strengths

There are some limitations to the study which would include the small sample size and length of the interview for each participant. Interviews were within a limited time frame due to GPs busy schedules. The study could have benefitted from comparing participants who were new graduates to GPs who have been in service for a greater length of time. However, the study's strength was in the in‐depth and data‐rich information obtained through open‐ended interviews with a diverse group of GPs.

## 5 CONCLUSION

This study explored how GPs respond to patients requesting imaging referrals and identified the underlying themes emerging from interviews around GPs' perspectives and experiences with patient expectations regarding imaging requests. The findings revealed that ‘impressive labels’, used in imaging reports, conveyed to patients a sense of seriousness, which in turn exacerbated patients' requests for them. GPs reported that some patients, especially those new to them, often felt entitled to receive imaging scans, with cost being of little concern. Furthermore, many patients who were experiencing anxiety placed significant value on radiological scans, and GPs acknowledged the importance of appeasing patient anxiety through the use of ‘therapeutic’ imaging while also practicing defensive medicine. Overall, this study highlights the complex dynamics involved in patient–GP interactions regarding imaging requests and suggests that developing evidence‐based strategies, perhaps through the professional bodies, might help manage the complexities surrounding such requests.

## CONFLICT OF INTEREST STATEMENT

The authors declare no conflict of interest.

## ETHICS STATEMENT

Institutional ethics approval was granted by the University of Sydney Human Research Ethics Committee (HREC) Project number: 2022/520. Approval period: August 15 2022 to 2026.

## Supporting information

Supporting information.

## Data Availability

The data that support the findings of this study are available from the corresponding author upon reasonable request.

## References

[1] Picano E , Pasanisi E , Brown J , Marwick TH . A gatekeeper for the gatekeeper: inappropriate referrals to stress echocardiography. Am Heart J. 2007;154(2):285‐290. 17643578 10.1016/j.ahj.2007.04.032

[2] Wilson IB , Dukes K , Greenfield S , Kaplan S , Hillman B . Patients' role in the use of radiology testing for common office practice complaints. Arch Intern Med. 2001;161(2):256‐263. 11176741 10.1001/archinte.161.2.256

[3] Beisecker AE , Beisecker TD . Using metaphors to characterize doctor–patient relationships: paternalism versus consumerism. Health Commun. 1993;5(1):41‐58.

[4] Huisman M , Joye S , Biltereyst D . Searching for health: doctor Google and the shifting dynamics of the middle‐aged and older adult patient–physician relationship and interaction. J Aging Health. 2020;32(9):998‐1007. 31517558 10.1177/0898264319873809

[5] Salinas J , Courtwright A . Autonomy and the “demanding encounter” in clinical neurology. Neurol Clin Pract. 2015;5(2):126‐131. 29443191 10.1212/CPJ.0000000000000106 PMC5764445

[6] Rogers WA . Whose autonomy? Which choice? A study of GPs' attitudes towards patient autonomy in the management of low back pain. Fam Pract. 2002;19(2):140‐145. 11906978 10.1093/fampra/19.2.140

[7] Gransjøen AM , Wiig S , Lysdahl KB , Hofmann BM . Barriers and facilitators for guideline adherence in diagnostic imaging: an explorative study of GPs' and radiologists' perspectives. BMC Health Serv Res. 2018;18(1):556. 30012130 10.1186/s12913-018-3372-7 PMC6048703

[8] Fenton J , Deyo RA . Patient self‐referral for radiologic screening tests: clinical and ethical concerns. J Am Board Fam Med. 2003;16(6):494‐501. 10.3122/jabfm.16.6.494 14963076

[9] Jutel A . “Dr. Google” and his predecessors. Diagnosis. 2017;4(2):87‐91. 29536917 10.1515/dx-2016-0045

[10] Fenton J , Kravitz RL , Jerant A , et al. Promoting patient‐centered counseling to reduce use of low‐value diagnostic tests: a randomized clinical trial. JAMA Intern Med. 2016;176(2):191‐197. 26640973 10.1001/jamainternmed.2015.6840

[11] Zigman Suchsland ML , Witwer E , Truitt AR , et al. Patient‐centered outcomes related to imaging testing in US primary care. J Am Coll Radiol. 2019;16(2):156‐163. 30482736 10.1016/j.jacr.2018.08.021 PMC7050575

[12] Thomas S , O'Loughlin K , Clarke J . Sonographers' communication in obstetrics: challenges to their professional role and practice in Australia. Australas J Ultrasound Med. 2020;23(2):129‐139. 34760592 10.1002/ajum.12184 PMC8411765

[13] Fenton J , Franks P , Feldman MD , et al. Impact of patient requests on provider‐perceived visit difficulty in primary care. J Gen Intern Med. 2015;30(2):214‐220. 25373836 10.1007/s11606-014-3082-8 PMC4314480

[14] DeJonckheere M , Vaughn LM . Semistructured interviewing in primary care research: a balance of relationship and rigour. Fam Med Commun Health. 2019;7(2):e000057. 10.1136/fmch-2018-000057 PMC6910737 32148704

[15] Downe‐Wamboldt B . Content analysis: method, applications, and issues. Health Care Women Int. 1992;13(3):313‐321. 1399871 10.1080/07399339209516006

[16] Chou L , Ranger TA , Peiris W , et al. Patients' perceived needs for medical services for non‐specific low back pain: a systematic scoping review. PLoS One. 2018;13(11):e0204885. 30408039 10.1371/journal.pone.0204885 PMC6224057

[17] Hoffman NY , Janus J , Destounis S , Logan‐Young W . When the patient asks for the results of her mammogram, how should the radiologist reply? Am J Roentgenol. 1994;162(3):597‐599. 8109504 10.2214/ajr.162.3.8109504

[18] Hofmann B , Lysdahl KB . Moral principles and medical practice: the role of patient autonomy in the extensive use of radiological services. J Med Ethics. 2008;34(6):446‐449. 18511617 10.1136/jme.2006.019307

[19] Griffith J , Monkman H , Borycki E , Kushniruk A . Physician experiences with perceived pressure to order diagnostic imaging services. Stud Health Technol Inform. 2015;218:20‐25. 26262521

[20] Epstein RM . Patient‐centered communication and diagnostic testing. Ann Fam Med. 2005;3(5):415‐421. 16189057 10.1370/afm.348 PMC1466928

[21] Walderhaug KE , Nyquist MK , Mjølstad BP . GP strategies to avoid imaging overuse. A qualitative study in Norwegian general practice. Scand J Prim Health Care. 2022;40(1):48‐56. 35188069 10.1080/02813432.2022.2036480 PMC9090343

[22] Artus M , van der Windt DA , Afolabi EK , et al. Management of shoulder pain by UK general practitioners (GPs): a national survey. BMJ Open. 2017;7(6):e015711. 10.1136/bmjopen-2016-015711 PMC5734284 28637737

[23] De Rosis S , Barsanti S . Patient satisfaction, e‐health and the evolution of the patient–general practitioner relationship: evidence from an Italian survey. Health Policy. 2016;120(11):1279‐1292. 27836231 10.1016/j.healthpol.2016.09.012

[24] Borracci RA , Manente D , Giorgi MA , Calderón G , Ciancio A , Doval HC . [Patients' preferences for information in health care decision‐making]. Medicina. 2012;72(5):393‐398. 23089115

[25] Nowell LS , Norris JM , White DE , Moules NJ . Thematic analysis: striving to meet the trustworthiness criteria. Int J Qual Methods. 2017;16(1):160940691773384.

[26] Hsieh H‐F , Shannon SE . Three approaches to qualitative content analysis. Qual Health Res. 2005;15(9):1277‐1288. 16204405 10.1177/1049732305276687

[27] Braman SS . Chronic cough due to acute bronchitis. Chest. 2006;129(1):95S‐103S. 16428698 10.1378/chest.129.1_suppl.95S PMC7094612

[28] Battié MC , Joshi AB , Gibbons LE . Degenerative disc disease: what is in a name? Spine. 2019;44(21):1523‐1529. 31135628 10.1097/BRS.0000000000003103

[29] Carlsen B , Aakvik A . Patient involvement in clinical decision making: the effect of GP attitude on patient satisfaction. Health Expect. 2006;9(2):148‐157. 16677194 10.1111/j.1369-7625.2006.00385.x PMC5060341

[30] Mendelson RM , Montgomery BD . Towards appropriate imaging: tips for practice. Aust Fam Physician. 2016;45(6):391‐395. 27622229

[31] Fenton JJ , Franks P , Feldman MD , et al. Impact of patient requests on provider‐perceived visit difficulty in primary care. J Gen Intern Med. 2014;30(2):214‐220. 25373836 10.1007/s11606-014-3082-8 PMC4314480

[32] Brodersen J , Schwartz LM , Heneghan C , O'Sullivan JW , Aronson JK , Woloshin S . Overdiagnosis: what it is and what it isn't. BMJ Evid Based Med. 2018;23(1):1‐3. 10.1136/ebmed-2017-110886 29367314

[33] Fenton JJ , Jerant A , Franks P , et al. Watchful waiting as a strategy to reduce low‐value spinal imaging: study protocol for a randomized trial. Trials. 2021;22(1):167. 33639993 10.1186/s13063-021-05106-x PMC7910785

[34] Hogue M‐CB , Doran E , Henry DA. A prompt to the web: the media and health information seeking behaviour. PLoS One 2012;7(4):e34314. 22509289 10.1371/journal.pone.0034314 PMC3317974

[35] Berg AO , Atkins D , Tierney W . Clinical practice guidelines in practice and education. J Gen Intern Med. 1997;12(S2):25‐33. 9127241 10.1046/j.1525-1497.12.s2.4.x PMC1497225

