# Supplementary material for: Patients' requests for radiological imaging: A qualitative study on general practitioners' perspectives
Source: Health Expect. 2023 Aug 16;26(6):2453–60. doi: 10.1111/hex.13849 (PMC10632629; doi:10.1111/hex.13849)
Supplement: Supplementary file 1 — Supporting information. [file HEX-26--s001.docx]

Doctors’ perspective on radiological requests by patients.

**Just a reminder that this interview is completely voluntary and at any time you wish to stop the interview please let me know. All data collected will be erased at your request. I would like to thank you for your time in agreeing to participate in this interview.**

**Interview Questions**

| **Questions** | **Probe** |
| --- | --- |
| ***Patient Taxonomy*** |  |
| Do you have patients that request radiological imaging from you? | Can you recall dealing with many in the past?  What sort of studies do patients generally request? How do you address these requests? |
| What are the presenting health concerns or circumstances that likely precede these requests? | Are there any specific characteristics of a patient that you have observed who would make these requests? For example a certain demographic type, personality trait, etc.? |
|  |  |
| ***Use of Radiology by doctor*** |  |
| Imaging has become an important part of patient care. How do you see the value in advancement of radiological technology, including high-tech imaging services such as multi-section computed tomography (CT), ultrasound, magnetic resonance (MR) imaging, and positron emission tomography (PET) aid in patients’ diagnosis? | In terms of accessibility to imaging, do you always have access to patients’ imaging history when they come to see you?  When patients’ previous images are not available, how does it impact you?  Could you please explain how you go about getting a patient’s past imaging history? |
| ***Interactions with radiologists*** |  |
| How would you describe your level of interactions with radiologists? Are they easy to contact and ask questions? | In terms of discussing limitations and merits of tests/procedures, are radiologists readily available to discuss these matters? Have you had any experiences that you can recall in the past where you needed to clarify information about a test/procedure? What was this experience like? |
| ***Guidelines to dealing with irrelevant requests*** |  |
| What steps do you take when patients present with an unnecessary imaging referral request? | Do you follow any particular guidelines in dealing with patients who are more “pushy” with their requests? |
| How pressured do you feel when needing to oblige patients’ requests? | What steps do you take to address unnecessary requests? |
| Could you please give reasons why doctors would oblige patients their requests? | Have you recently had any experiences with this situation? |
